# Experiences of being a family caregiver to a patient treated for oesophageal cancer—1 year after surgery

**DOI:** 10.1007/s00520-021-06501-5

**Published:** 2021-08-19

**Authors:** Cecilia H. Ringborg, Anna Schandl, Yvonne Wengström, Pernilla Lagergren

**Affiliations:** 1 Surgical Care Science, Department of Molecular Medicine and Surgery, Karolinska Institutet, Karolinska University Hospital, 171 76 Stockholm, Sweden; 2 Department of Anaesthesiology and Intensive Care, Södersjukhuset, Stockholm, Sweden; 3 Theme Cancer, Karolinska University Hospital, Stockholm, Sweden; 4 Department of Neurobiology, Care Science and Society, Division of Nursing, Karolinska Institutet, Stockholm, Sweden; 5 Department of Surgery and Cancer, Imperial College London, London, UK; 6 Surgical Care Science, Department of Molecular Medicine and Surgery, Karolinska Institutet, Retzius väg 13a, 171 65 Solna, Sweden

**Keywords:** Cancer survivorship, Psychosocial aspects, Family perspectives, Qualitative study

## Abstract

**Purpose:**

There is a need to put family caregivers on the cancer survivorship research agenda. Therefore, the aim of this is study is to explore the experiences of being a family caregiver to a patient treated for oesophageal cancer.

**Method:**

This qualitative study was based on the ongoing nationwide and prospective Oesophageal Surgery on Cancer patients – Adaptation and Recovery study (OSCAR) including patients surgically treated for oesophageal cancer in Sweden and their closest family caregiver. One year after the patient’s surgery, each family caregiver received a self-report questionnaire kit to fill in. For the purpose of this study, the responses to the open-ended question “Is there anything else you would like to share?” were used and analysed by conducting thematic analysis.

**Results:**

In total, 112 responses to the open-ended question were transcribed and analysed. The text rendered three themes:

***Discontinued support from healthcare***—mostly a positive experience before surgery and in the acute survivorship phase. However, after discharge from the hospital, the family caregiver felt as though they were left alone, fully responsible for the patient’s care.

***A changed life***—unprepared for life-changing situation after the patient received the cancer diagnosis. A feeling that nothing will ever be the same and like your sense of self is lost.

***Psychological distress***—was described as a feeling of being alone. Family caregivers felt invisible and no longer important to family and friends. The patient was the one that mattered.

**Conclusion:**

This study indicates that patients and family caregivers would benefit from a more family-centred healthcare, where the patients’ as well as the caregivers’ perspectives would be acknowledged.

## Introduction

A cancer diagnosis has not only a large impact on the patient but also affects the whole family. Being a family caregiver to a patient with cancer can be burdensome and emotionally draining [1]. Family caregivers of patients with oesophageal cancer have been shown to have an increased risk of long-lasting emotional distress [2, 3]. Oesophageal cancer carries a poor prognosis with an overall survival of 15% [4] and a 5-year survival rate of 30–55% for curatively treated patients [5]. The curative treatment for oesophageal cancer includes an extensive surgical procedure, most often in combination with neoadjuvant chemotherapy (with or without radiotherapy). Sometimes the surgery is followed by adjuvant therapy [6]. The survivors of oesophageal cancer often suffer from a reduced health-related quality of life (HRQL) in both the short and long term. Common problems in the years after surgery are eating difficulties, fatigue, reflux, and anxiety [7–9].

The experience of survivorship transition is highly individual and may vary in time. In the paper of Wood (2017), the transition is described as a concept including different attributes, antecedents, and consequences. These factors include both physical and psychosocial aspects as well as socioeconomic and cultural aspects. In order to target nursing interventions, we need to understand this concept so that we can identify what needs patients have that need to be targeted to support the transition [10]. Family caregivers to patients with cancer are a part of the survivorship experience [11]. However, most studies within this research field focus on patients’ survivorship transition and not on how family caregivers experience this journey. To be able to improve the survivorship for survivors of oesophageal cancer their transition, there is a need to gain understanding from the perspective of the family caregivers. Therefore, by using the questionnaire item “Is there anything else you would like to share?” we aimed to describe the experience of being a family caregiver to a patient treated for oesophageal cancer.

## Methods

### Study design, participants, and data source

This qualitative study was part of the ongoing nationwide and prospective Oesophageal Surgery on Cancer patients – Adaptation and Recovery study (OSCAR) including patients surgically treated for oesophageal cancer in Sweden and their closest family caregiver. OSCAR follows the ethical guidelines within the Declaration of Helsinki and was ethically approved in June 2013. A detailed description of the OSCAR study is presented elsewhere [12]. In brief, eligible patients were identified through a collaboration with all pathology departments in Sweden. One year after surgery, these patients were contacted by a project coordinator. At this time, the closest family caregiver was identified by the patient and asked to participate in the study by the project coordinator. After informed consent was obtained, the family caregiver received a questionnaire kit including several validated questionnaires and open-ended questions assessing their current life situation to fill in and return by post.

For this study, data from family caregivers who were included in the study between January 1, 2014, and August 30, 2019, who submitted a response to the open-ended question in the questionnaire “Is there anything else you would like to share?” were used for the analysis. All handwritten responses were scanned and transcribed verbatim into a separate document.

### Data analysis

A reflective thematic analysis was conducted [13] using all responses to the open-ended question. The first step in the analysis was familiarisation of the text where the researchers were required to read the transcribed text several times. Thereafter, codes were generated from the text. Codes that recurred frequently were grouped together and themes were developed. By using reflexive thematic analysis, the researchers could go back to the text, changing codes and themes reflectively during the analysis process [14]. Two researchers (CHR, AS) analysed the text separately. Both researchers were registered nurses with research experience of patients treated for oesophageal cancer and their family caregivers. Any disagreement was discussed until a consensus was reached. The analysis was also triangulated and discussed with the Surgical Care Science patient research partnership group within OSCAR to make sure that the results reflected the family caregivers’ experiences. The patient research partnership group included both patients surgically treated for oesophageal cancer and family caregivers.

## Results

### Participants

**Fig. 1:**
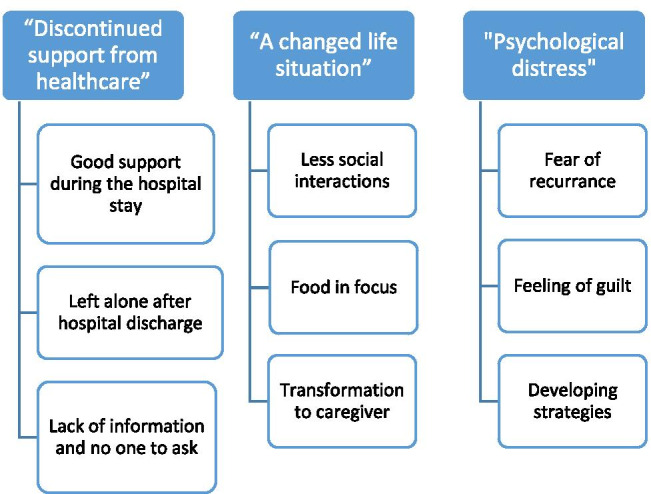
An overview of the results of the thematic analysis (themes and a brief description)

From the 238 family caregivers who were contacted, 112 responded to the open-ended question. The mean age of the family caregivers was 62 years and the majority of the family caregivers were women (87%). Most of the family caregivers were partners of the patients (75%), 11% were children and 14% were friends, siblings, or another relation to the patient. The transcribed text rendered three themes: (1) discontinued support from healthcare; (2) a changed life situation, and (3) psychological distress (see an overview of the results in Fig. 1).

### “Discontinued support from healthcare”

Family caregivers emphasised their trust and belief in cancer care. During the hospital stay, the caregivers perceived that the patient and the family caregiver were well taken care of. They were pleased with the pre-operative information provided about the treatment and the surgical procedure. However, there was a vast amount of information, and there was difficulty recalling some details. Therefore, it was suggested by the family caregivers that both the patient and the family caregiver should be present in the medical appointments at the hospital to receive the same information to ensure that nothing important was forgotten. Another suggestion to ensure that nothing was forgotten was to keep a diary during the hospital stay, especially during the time spent in intensive care. The diary could be used for both family caregivers and patients after hospital discharge to recall what happened during the hospital stay. When being in contact with healthcare staff, it was perceived to be difficult to ask the correct questions at the right time. The questions did not present themselves easily during the appointments. A family caregiver expressed that:

Sometimes, it is like, you do not come up with the right questions to ask, they emerge continuously. As a family caregiver you might need help to ask them, it is a bit like being in a vacuum.

The family caregivers were satisfied with the patients’ medical treatment and supportive care during the hospital stay. When the patient received the cancer diagnosis and during treatment, the contact with healthcare was expressed as great and the information transmission was well-functioning. A small number of the family caregivers emphasised the importance of having a close continuous interaction with the contact nurses. They especially appreciated always having someone to contact for questions. However, after the patients’ discharge, the majority of the family caregivers experienced a feeling of being left alone without support and did not know where, or who to turn to with questions. Their experience was that the patients heavily relied on them for support, which could be burdensome and at a high cost to themselves. Some family caregivers also wished that they had the opportunity to take time off work to take care of their family member.

There is no coherent follow-up for the patient; the initiative is with us, as well as the responsibility.

On discharge from the hospital, most patients were still in great need of supportive care and the family caregivers stated that a rehabilitation stay where the patients’ functional status could have been optimised before coming home would have facilitated the recovery process and could have reduced the burden for the caregiver. The family caregivers expressed that they did not know how to care for the patient at home and lacked information on how to fulfil the patient’s fundamental care needs in the best possible way. They would have appreciated practical advice on, e.g., nutrition and how to exercise.

I thought the lack of guidance of nutrition and physical activity after the surgery was shocking!

Moreover, they lacked an identified contact in healthcare, who they could turn to for support, and contact for more information if needed.

The family caregivers suggested a follow-up meeting just for family caregivers where they could talk about their experiences, ask questions, and receive advice and support on how to move on with life.

### “A changed life situation”

The family caregivers stated that their life changed after the patients’ surgery. They were unprepared for this change. They described that life was not as active as before, with less social interaction. They also referred the loss of social interactions to the patient’s changed food intake and altered general condition, being much more tired as one of the major reasons for being invited to fewer social events. They worried about the patient not gaining enough weight or having difficulties eating. To prevent weight loss, many family caregivers served the patient smaller portions of more caloric or protein-rich food. Consequently, the family caregivers also gained weight unintentionally. The family caregivers also stated that they strived to make the food situation as pleasant as possible and tried to avoid conflicts while eating.

Fatigue, another common consequence of cancer treatment, contributed to the patient’s lack of strength or reduced ability to take part in social interactions. Inevitably, these problems also affected the family caregivers. One family caregiver stated:

You get lonelier than before. The person that is sick struggles with eating problems, it is not much fun to invite someone when not being able to eat like before and who wants to take a nap from time to time. That is not very nice.

All the positive aspects with food and eating disappeared with the disease.

After surgery, the family caregivers experienced that they had to do most of the daily duties at home since the patient no longer had the strength to contribute. Previously, taking care of the household had been the responsibility of both patient and the partner. However, after the treatment, the patient’s lack of energy changed the balance of responsibility. Things were not like before. The changed life included being a full-time caregiver. Before treatment, the patient was capable of taking care of himself /herself but after treatment, the relationship transformed and the family member became a caregiver.

You feel like your personal self is eradicated. Your life purpose has become to take care of your family member.

Some family caregivers said that pre-operative, as well as post-operative, information about treatment side effects and the potential life changes would have allowed them time to prepare for the life change that the surgery might entail.

### “Psychological distress”

Worry about the patient’s disease and potential tumour recurrence was frequently experienced and perceived as physiologically draining. Anxiety about the risk of recurrence and death was common among many family caregivers and caused them sleep problems.

I thought he would die, I hope no one ever has to experience this.

Sometimes, even though the family caregivers wanted to do their best, they lost the strength to take care of the patient. Not being able to take care of the patient was seen as a failure and evoked feelings of guilt.

I have carried a bunch of guilty feelings that I didn’t apply for sick-leave from work so that I could have been at home when my husband was feeling bad after the treatment. It is mostly my feeling, but no one ever asked how I was doing and if I needed to be at home. I wanted to be strong and maintain a normal life. Afterwards, I would have acted differently.

The family caregivers also struggled with the feeling that they were now solely identified as family caregivers and not as the person they were before the patients were diagnosed with oesophageal cancer. They felt that they had lost their value as an individual and they were unnoticed, e.g. they were never asked how they were doing. It was expressed as “being invisible” to other family members and friends and experiencing a “loss of identity”. Everyone was concerned about the patient’s, and not the caregivers’, wellbeing.

… you live alone in your mind because no one can understand how you feel and how the disease affects you. Not even yourself.

Nothing will ever be the same, neither for the patient and nor for you.

The family caregivers described how they had developed strategies to cope with the situation. They recommended others in a similar situation, to be open-minded and to try to have an open conversation with the patient about the disease, death, and their future life together. They stressed the importance of trying to lead as normal a life as possible. One piece of advice given was to try to stay positive.

Try to support each other through the good days as well as the bad days.

Do not give up too early! Make the most of the positive things! That helps at the moment.

It was suggested that you should not be too hard on yourself and to take care of your own feelings. As family caregivers, it was suggested you allow yourself to have a break now and then.

## Discussion

In this study, we explored the experiences of family caregivers to patients 1 year after surgery for oesophageal cancer by analysing data from an open-ended question. Overall, the family caregivers seemed satisfied with the medical treatment and supportive care which the patient received during the hospital stay. However, after hospital discharge, they felt that they had to carry a large responsibility for the patients’ care. They lacked support and practical advice on how to, in the best possible way, take care of the patient. This would have been beneficial for the patients’ trajectory of recovery and their own wellbeing. The extensive surgery with its consequences on normal life renders a completely new life situation involving altered roles and responsibilities which is challenging. This is in line with what is described in the survivorship transition by Wood (2017). It seems that patients and their family caregivers have similar experiences with new needs, distress, and their adaptation to a new identity and a new life situation. In addition, regarding the results of the current study, there seems to be unmet needs regarding the consequences for both family caregivers but also for patients in the transition to cancer survivorship.

In this study, family caregivers seemed to take on a large responsibility for the patient’s care when the healthcare did not cover the patient’s needs. The family caregivers saw themselves as responsible for the patient’s contact with the healthcare setting, in order to confirm that the patient’s needs were fulfilled. This resulted in less social interaction and a feeling of losing your sense of self. In one American cohort study including 111 caregivers to patients with cancer, it was found that family caregivers often set aside their own work to prioritise taking care of the patient. Some family caregivers restricted their work time and others even completely stopped working in order to focus on taking care of the family member [15]. In a Swedish interview study [16], many family caregivers of patients with cancer expressed that they were “working caregivers” with a large responsibility of taking care of daily home activities, rather than family members. They also described a loss of identity and considered themselves as project managers, taking care of the patient and his/her needs [16], similar to what was found in our study.

One cohort study including 47 couples made up of oesophageal cancer patients and family caregivers found that 33% of the family caregivers reported a high caregiver burden for as many as 3 years after the patients’ curative treatment was completed [17]. This suggests that the family caregiver burden persists for a long time after the patient’s treatment has been completed. The burden among family caregivers and the fear of tumour recurrence might result in new-onset psychological problems among family caregivers. In a previous study, an association was found between patients with cancer’s psychological distress and their family caregivers’ psychological distress. However, the level of distress depended on the phase of the illness. During the survivorship phase, the fear of tumour recurrence was predominant among family caregivers [18].

The lack of healthcare support after hospital discharge for the family caregivers and the patients was repeatedly stated in our data. There seems to be a need to individualise support after treatment for oesophageal cancer, including the psychosocial aspects, the burden of responsibility, and risk of psychological distress so that the emotional impact of caregiving can be reduced. One way of doing that could be to structure long-term follow-up initiated by the personal contact nurses. In the current study, having a contact nurse was equal to being assured that the family caregivers had someone to turn to when needed. In Sweden, all patients receive a contact nurse who follows the patient throughout the care pathway up to 3 years postoperatively and acts as their primary contact to healthcare [19]. However, as being expressed in the current study, there seem to be some problems regarding information and follow-up at 1 year after treatment. It has been demonstrated in a previous systematic review that the lack of information, both regarding illness and treatment but also care information, is the most prominent unmet need in family caregivers [20]. The healthcare professionals might be unaware about what the family members are going through during the survivorship transition phase. Most follow-up appointments focus on the patient’s wellbeing and not the family caregivers’. Healthcare in the municipality might take into consideration that caregivers need to share their concerns with the healthcare to enhance their knowledge and skills on caring. By that, they can get the support if particular vulnerable or with lack of experience handling the caregiver function.

Holistic support for oesophageal cancer survivors and family caregivers has been seen as a challenge in previous research and is in line with the ongoing development of more person-centred care [21, 22]. This type of support is needed to adjust for the social, psychological, and physical consequences of oesophageal cancer surgery [23]. Another suggestion is to include individual (long-term) survivorship care plans (ISP) for the patient, which includes their life situation and involve the family caregivers in the planning [24]. By using an ISP, all perspectives might be taken into consideration such as optimising the patient’s medical treatment, supportive care, rehabilitation, and life situation. In this way, it would be evident for the family caregivers that they were not the only ones in charge of the patients’ rehabilitation needs. The next step for future research would be to develop, implement, and evaluate supportive care interventions for patients and family caregivers in the survivorship phase after oesophageal cancer. One suggestion to help the family caregivers to reduce their feeling of loneliness and the feeling that no one understands could be an intervention with a digital forum. For example, an application with information about the disease, treatment, and side effects but also the function to interact with others. This could be used to help the family caregivers to meet with other family caregivers. In this way, they could exchange experiences and information, but also feel an affinity in a context.

The nationwide design of the study with a high response rate contributes to the transferability of the results. Having two researchers conducting the analysis separately and then validating the findings with a patient/family caregiver research partner improves the credibility of the results. Regarding confirmability, the open-ended question was optional to fill in and the family caregivers wrote the text in their own words, which minimises the risk of researcher bias. The downside with open-ended questions is that it precludes asking follow-up questions and thereby limits the possibility of reaching deeper into the narrative.

## Conclusion

Family caregivers of oesophageal cancer patients experience discontinued support from healthcare, a changed life situation, and psychological distress after the patients’ surgery. This resulted in several challenges among family caregivers. These results point out that there are deficits in the implementation of rehabilitation and follow-up during the first year of survivorship for patients treated for oesophageal cancer. It also shows that there is an unmet need to support the family caregivers. There is a need to reduce the burden to the family caregivers and contribute with support during the patient’s survivorship care.

## Data Availability

Upon request.

## Code availability

Upon request.
